# Helminth parasites infecting feral pigeons *(Columba livia)* in Al Ain City, United Arab Emirates

**DOI:** 10.2478/jvetres-2026-0009

**Published:** 2026-02-24

**Authors:** Yassir Sulieman, Nighat Perveen, Mohammad Ali Al-Deeb, Theerakamol Pengsakul

**Affiliations:** Biology Department, College of Science, United Arab Emirates University, PO Box 15551, Al Ain, United Arab Emirates; Department of Zoology, Faculty of Science and Technology, Shendi University, PO Box 142 or 143, Shendi, River Nile State, 11111, Sudan; Health and Environmental Research Center, Faculty of Environmental Management, Prince of Songkla University, Hat Yai, Songkhla, 90110, Thailand

**Keywords:** *Columba livia*, helminth parasites, lifestyle differences, sex differences, microscopy

## Abstract

**Introduction:**

*Columba livia*, the common pigeon, is the most abundant free-ranging avian species in the United Arab Emirates (UAE). Despite the pigeon’s ecological prominence, data on helminth parasites infecting this host remain undocumented in the region. This investigation examined helminth infections in both wild and domestic pigeon populations in the city of Al Ain to establish baseline parasitological records.

**Material and Methods:**

A cross-sectional survey conducted between August 2023 and October 2024 analysed 100 adult pigeons obtained through trapping and local market acquisition. The helminth parasites recovered were morphologically identified, and parasitological indicators were determined including infection prevalence and intensity.

**Results:**

Helminths were detected in 69% of examined birds, with a mean infection intensity of 6.4 parasites per host. Seven helminth species were identified: three cestodes (*Raillietina echinobothrida, Raillietina tetragona* and *Cotugnia digonopora*), three nematodes (*Ascaridia galli, Dispharagus nasutus* and *Gongylonema* sp.), and one trematode (*Brachylaima* sp.). *Raillietina echinobothrida* (57%) and *C. digonopora* (21%) were the most prevalent. Key epidemiological findings showed male pigeons had significantly higher infection intensity (5.1 *vs* 3.5 parasites/bird) than females, though prevalence differences were not significant (71.4% *vs* 65.9%). Crucially, feral pigeons exhibited a dramatically higher infection prevalence (92%) than domestic pigeons (46%). However, infection intensity differences between feral (2.7) and domestic (2.3) birds were not significant.

**Conclusion:**

As the first helminthological assessment of UAE pigeons, this study documents seven new regional parasite records and underscores the need for continued surveillance to assess potential zoonotic and ecological risks in urban ecosystems.

## Introduction

Pigeons (Aves: Columbidae) are globally distributed birds closely associated with urban environments (22). Certain species have been domesticated and utilised for food, ornamental purposes, entertainment, and more recently, as laboratory models (10, 37). However, most pigeons live free, constructing nests on buildings (particularly under eaves), in public parks, woodlands and rocky outcrops (16). Their prolific droppings soil many man-made structures, leading to aesthetic degradation, structural damage and material deterioration (19). Additionally, pigeons are recognised as agricultural pests, causing significant crop damage to grains and seeds (22).

This species is susceptible to diverse pathogen infections, including protozoans (*e.g. Trichomonas gallinae, Eimeria columbae, Toxoplasma gondii* and *Cryptosporidium* spp.), nematodes (*e.g. Capillaria* spp., *Syngamus* spp., *Ascaridia* spp. and *Hadjelia truncata*), platyhelminths (*e.g. Raillietina* spp., *Hymenolepis* spp. and *Echinostoma* spp.) and ectoparasites such as ticks, mites and lice (2, 27, 32). The epidemiology of these parasites is shaped by host-related factors (*e.g*. age, sex and foraging behaviour) and parasite traits such as environmental resilience and transmission dynamics (44). Investigating the risk factors and their association with parasite prevalence is essential for designing targeted prevention and control measures.

Pigeons’ capacity for long-distance flight and frequent interactions with diverse avian populations elevate their potential to transmit parasites across species barriers, posing risks to both wildlife and humans (20, 35). Such parasite transmission threatens poultry and economically valuable avian species, often resulting in weakened immunity, impaired development, reduced body condition and declining health (11). In severe cases, infestations can escalate to fatal outcomes if left untreated (47).

*Columba livia*, known as the common pigeon, is the predominant free-living avian species across the United Arab Emirates (UAE), thriving in urban habitats such as public parks, commercial markets and mosque courtyards. Listed as Least Concern (LC) on the IUCN Red List (21), this species sustains robust populations in the city of Al Ain, where substantial feral flocks persist without human management. Despite their ecological prominence, parasitic infections in these birds remain understudied. This study addresses this knowledge gap by assessing the prevalence and infection intensity of helminth parasites across wild and domestic pigeon populations.

## Material and Methods

### Study area

Al Ain, the second-largest city in the Abu Dhabi Emirate, UAE (24°12′27″N, 55°44′4l″E), has an estimated population of 666,000 residents. Located approximately 160 km east of Abu Dhabi and 120 km south of Dubai, the city experiences a hot desert climate characterised by extremely high summer temperatures, mild winters and an average annual rainfall of 96 mm, with relative humidity averages around 60%.

### Sampling and parasite identification

Between August 2023 and October 2024, a total of 100 pigeons (*C. livia*) were collected, comprising 50 domestic and 50 feral individuals. Domestic birds were sourced from Al Ain’s local pet market, while feral pigeons were captured using traps placed across public squares, parks and market areas. All specimens were transported in ventilated boxes to the laboratory at the Department of Biology, UAE University, where they were humanely euthanised using diethyl ether.

During necropsy, birds were clinically assessed, sexed and dissected. Internal organs (the oesophagus, crop, proventriculus, gizzard, duodenum, ileum, rectum, pancreas, liver, lungs and heart) were excised, placed in Petri dishes containing 0.9% saline for 10 min and examined under a stereomicroscope. Detected parasites (cestodes, trematodes and nematodes) were isolated using fine forceps or Pasteur pipettes, counted and preserved in 70% ethanol containing 5% glycerin.

Cestodes and trematodes were stained with acetocarmine, dehydrated in a graded ethanol series (70%–100%), cleared in xylol-methyl salicylate and permanently mounted on slides. Nematodes were examined as temporary wet mounts. Identification to genus or species level was carried out under a light microscope using standard taxonomic keys (5, 25, 45). Photomicrographs were taken using a Samsung SM-S918B/DS camera (Seoul, South Korea) adapted to an Olympus CX33 light microscope (Tokyo, Japan).

### Ethical considerations

Sampling was conducted under the supervision of Barari Natural Resources, Environment Agency–Abu Dhabi, UAE (Scientific research no-objection certificate No. 200100118). All animal handling procedures adhered to the guidelines of the Ethics Committee on Animal Use, UAE University (approval code: ERA_2024_5106).

### Data analysis

Data were analysed using SPSS 29.0 (IBM, Armonk, NY, USA). Infection prevalence (*P*) and mean intensity (*MI*) were calculated as *P = n/Z × 100* and *MI = N/n*, where *n* = infected birds, *Z* = total birds examined and *N* = total parasites (9). Prevalence differences were assessed *via* chi-squared tests, and intensity variations were analysed using paired *t*-tests. Significance was set at P-value < 0.05.

## Results

### Prevalence, intensity and diversity of infection

**Fig. 1.**
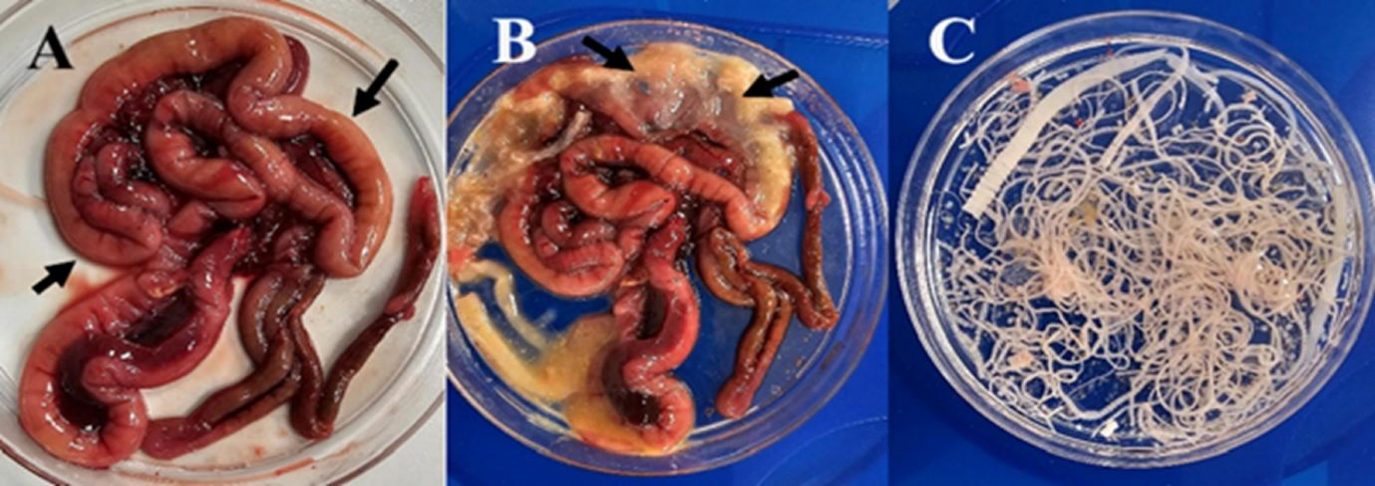
Intestinal tract from *Columbia livia* collected in Al Ain, UAE. A – upper intestine showing cestode infection (yellowish white); B – opened upper intestine displaying cestodes; C – cestodes recovered from a heavily infected *C. livia*

**Fig. 2.**
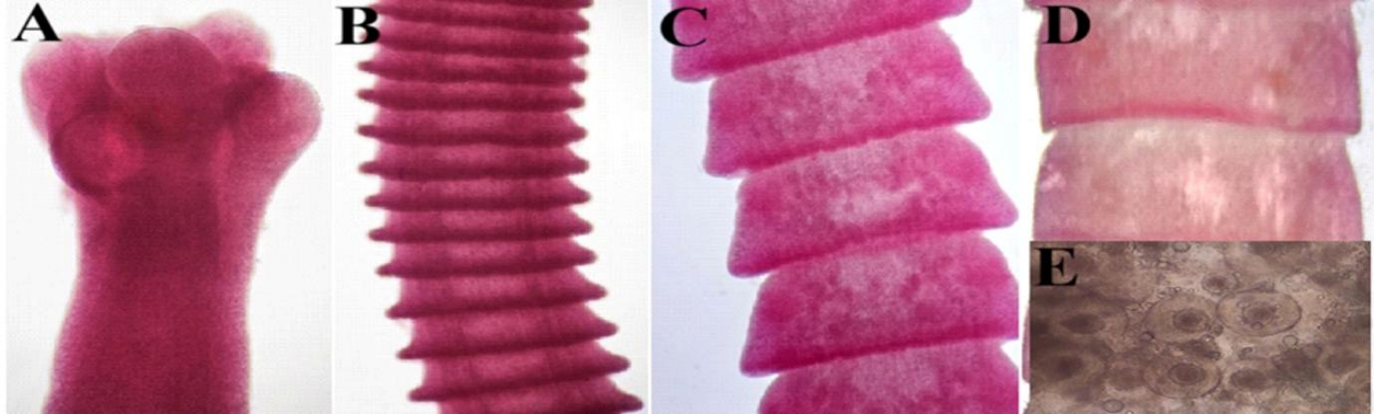
Light micrographs of *Cotugnia digonopora* specimens obtained from *Columbia livia* in Al Ain, UAE. A – scolex (100×); B – immature proglottid series (100×); C – mature proglottids (100×); D – gravid proglottids (100×); E – eggs (400×)

**Fig. 3.**
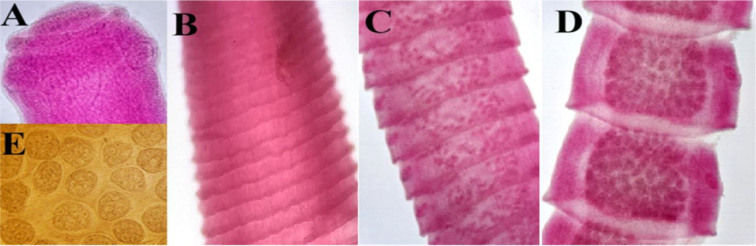
Light micrographs of *Raillietina echinobothrida* specimens obtained from *Columbia livia* in Al Ain, UAE. A – scolex (100×); B –immature proglottid series (100×); C – mature proglottids (100×); D – gravid proglottids (100×); E – eggs (400×)

**Fig. 4.**
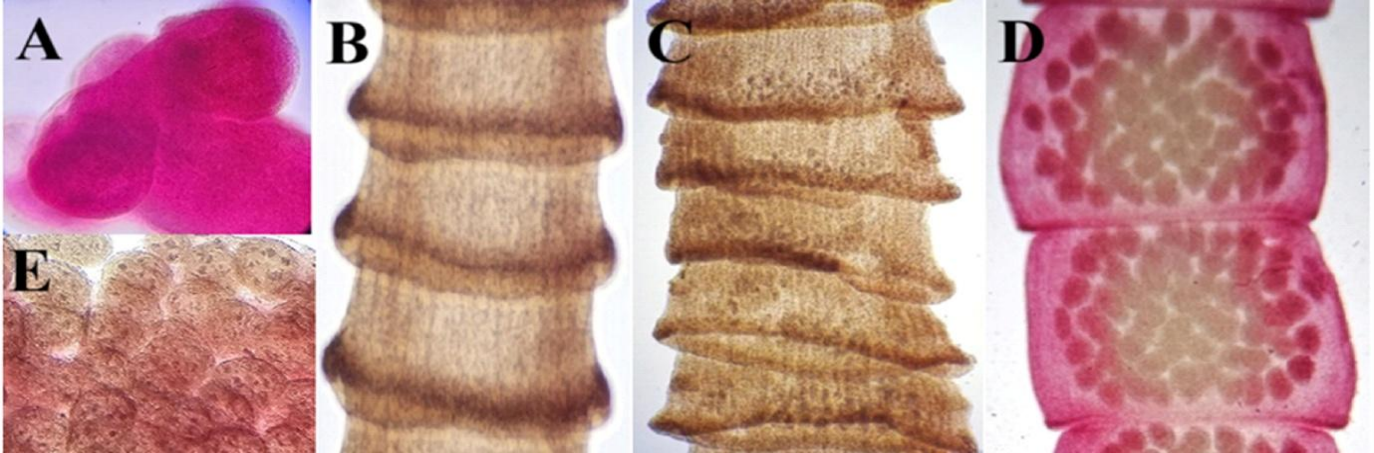
Light micrographs of *Raillietina tetragona* specimens obtained from *Columbia livia* in Al Ain, UAE. A – scolex (100×); B – immature proglottid series (100×); C – mature proglottids (100×); D – gravid proglottids (100×); E – eggs (400×)

**Fig. 5.**
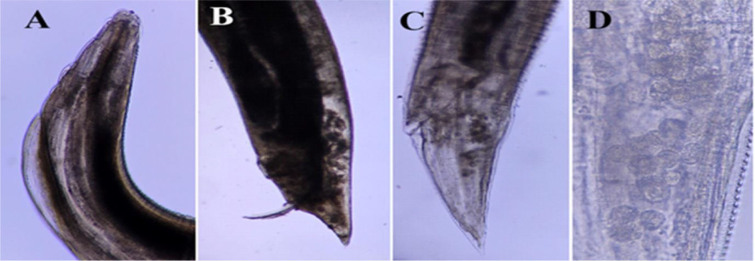
Light micrographs of *Ascaridia galli* specimens obtained from *Columbia livia* in Al Ain, UAE. A – male and female anterior end (300×); B – male posterior end (120×); C – female posterior end (300×); D – uterus containing eggs (1,200×)

**Fig. 6.**
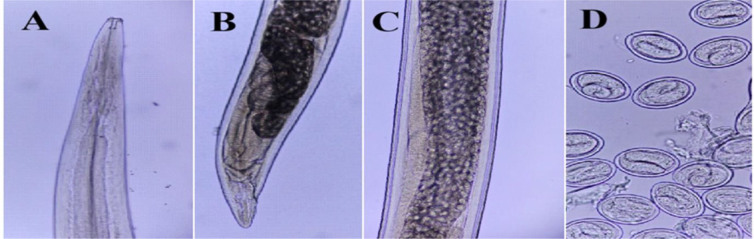
Light micrographs of *Dispharagus nasutus* specimens obtained from *Columbia livia* in Al Ain, UAE. A – male and female anterior end (300×); B – female posterior end (300×); C – uterus filled with eggs (300×); D – eggs (1,200×)

**Fig. 7.**
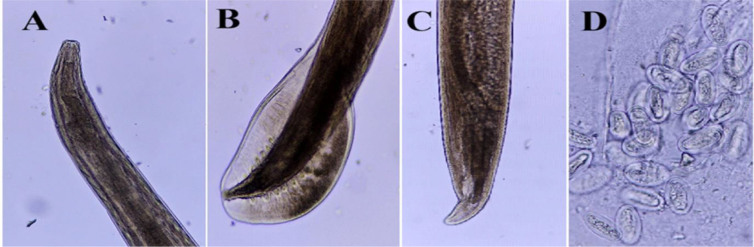
Light micrographs of *Gongylonema* sp. specimens obtained from *Columbia livia* in Al Ain, UAE. A – male and female anterior end (300×); B – male posterior end (300×); C – female posterior end (300×); D – eggs (1,200×)

**Fig. 8.**
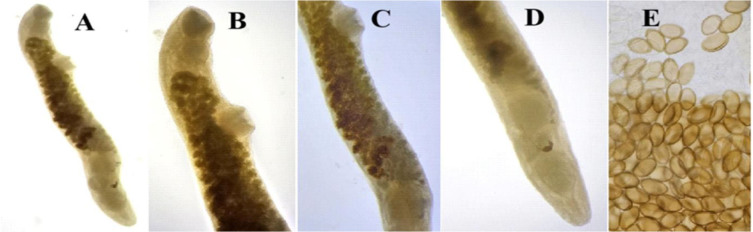
Light micrographs of *Brachylaima* sp. specimens obtained from *Columbia livia* in Al Ain, UAE. A – whole mount (40×); B – anterior body (100×); C – central body (100×); D – posterior body (100×); E – eggs (400×)

Of the 100 adult pigeons (*C. livia*) examined (56 males and 44 females), 69 were infected with at least one helminth species, with a mean infection intensity of 6.4 parasites per bird. Clinical symptoms observed in infected individuals included weight loss, diarrhoea and intestinal wall inflammation. Seven helminth species were identified in the internal organs: three cestodes (*Raillietina echinobothrida, R. tetragona* and *Cotugnia digonopora* (Figs 1–4), three nematodes (*Ascaridia galli, Dispharagus nasutus* and *Gongylonema* sp. (Figs 5–7) and one trematode (*Brachylaima* sp. (Fig. 8). The cestode *R. echinobothrida* was the most prevalent species, followed by *C. digonopora*, while the nematodes *D. nasutus* and *Gongylonema* sp. were the least common (Table 1). However, *Gongylonema* sp. exhibited the highest infection intensity, in contrast to *C. digonopora*, which had the lowest (Table 1). Single-species infections were detected in 42% of pigeons, while 27% harboured mixed infections involving two helminth species.

**Table 1.** Helminth infection parameters and infection sites identified in pigeons (n = 100) sampled in Al Ain between August 2023 and October 2024

Helminth species	Number of infected birds	Prevalence %	Mean intensity ± SE	Intensity range	Infection site
Cestoda					
*Raillietina echinobothrida*	57	57	4.5 ± 0.5	1–17	Duodenum and ileum
*R. tetragona*	9	9	3.6 ± 0.9	1–10	Duodenum and ileum
*Cotugnia digonopora*	21	21	3.5 ± 0.4	1–8	Duodenum and ileum
Trematoda					
*Brachylaima* sp.	5	5	7.4 ± 1.7	3–12	Rectum
Nematoda					
*Ascaridia galli*	5	5	4 ± 0.6	2–5	Ileum
*Dispharagus nasutus*	1	1	4	4	Gizzard
*Gongylonema* sp.	1	1	18	18	Gizzard
Overall	69	69	6.4 ± 0.3	1–18	

### Infection parameters by sex

Although male pigeons showed a higher prevalence of infection (71.4%) than females (65.9%), this difference was not statistically significant (P-value > 0.05). However, males exhibited significantly greater infection intensity compared to females (P-value < 0.05) (Tables 2 and 3).

**Table 2.** Prevalence of pigeon helminth infections and comparative analysis by bird sex and lifestyle (n = 100) for sample pigeons from Al Ain between August 2023 and October 2024

Helminth species	Prevalence (%)							
	Male (n = 56)	Female (n = 44)	*χ^2^*	P-value	Feral (n = 50)	Domestic (n = 50)	*χ^2^*	P-value
Cestoda								
*Raillietina echinobothrida*	58.9	54.5	9.3	0.8	84	30	15.3	0.3
*R. tetragona*	10.7	6.8	3.5	0.5	16	2	9	0.06
*Cotugnia digonopora*	28.6	11.4	6.6	0.4	28	14	11.8	0.08
Trematoda								
*Brachylaima* sp.	8.9	0	-	-	10	0	-	-
Nematoda								
*Ascaridia galli*	3.6	6.8	2.2	0.3	6	4	2.2	0.3
*Dispharagus nasutus*	0	2.3	-	-	0	2	-	-
*Gongylonema* sp.	1.8	0	-	-	2	0	-	-
Overall	71.4	65.9	0.4	0.6	92	46	24.7	0.001

**Table 3.** Intensity of pigeon helminth infections and comparative analysis by bird sex and lifestyle (n = 100) for sample pigeons from Al Ain between August 2023 and October 2024

Helminth species	Mean intensity ± SE							
	Male (n = 56)	Female (n = 44)	*t*	P-value	Feral (n = 50)	Domestic (n = 50)	*t*	P-value
Cestoda								
*Raillietina echinobothrida*	5.2 ± 0.8	3.5 ± 0.5	1.6	0.004	5.4 ± 0.7	2.1 ± 0.4	–2.9	0.001
*R. tetragona*	4.3 ± 1.2	2 ± 0.6	1.3	0.2	3.9 ± 0.9	1	–1.0	0.2
*Cotugnia digonopora*	3.4 ± 0.4	3.8 ± 0.9	0.04	0.6	4.1 ± 0.5	2.3 ± 0.7	–2.3	0.9
Trematoda								
*Brachylaima* sp.	7.4 ± 1.7	-	-	-	7.4 ± 1.7	-	-	-
Nematoda								
*Ascaridia galli*	4 ± 1	4 ± 1	0	0.5	4.3 ± 0.7	3.5 ± 1.5	-0.5	0.12
*Dispharagus nasutus*	0	4	-	-	0	4	-	-
*Gongylonema* sp.	18	0	-	-	18	0	-	-
Overall	5.1 ± 0.8	3.5 ± 0.4	2.1	0.02	2.7 ± 0.3	2.3 ± 0.3	–1.2	0.12

### Infection parameters by lifestyle (feral *vs* domestic)

Feral pigeons exhibited a markedly higher infection prevalence (92%) than domestic pigeons (46%), a difference that was statistically significant (P-value < 0.05). Feral birds also showed higher infection intensity than domestic individuals, although this difference was not statistically significant (P-value > 0.05) (Tables 2 and 3).

## Discussion

To the authors’ knowledge, this study represents the first documented investigation of helminth parasites in Al Ain’s pigeon populations, addressing a critical gap in regional parasitological research. The observed overall helminth prevalence of 69% aligns with reports from Tanzania of 79.5% (32), Botswana of 92% (33) and India of 100% (8), but contrasts with lower rates in Egypt of 11.8% (14), Turkey of 29% (17) and Iran of 42% (30). On the other hand, infection intensity (6.4 helminths per host) was markedly lower than Nigeria’s reported 15.4 helminths per host (2), although limited comparable data preclude broader conclusions. The dominance of cestodes likely reflects the abundance of arthropod intermediate hosts (*e.g*. insects) in urban ecosystems. Disparities in prevalence and intensity across regions may stem from multiple factors, including methodological variations in parasite detection, ecological availability of intermediate hosts or vectors and local climatic influences (31, 44).

In the current study, pigeons exhibiting clinical signs of diarrhoea and weight loss carried higher parasitic loads. These findings align with prior studies suggesting that parasitic infections compromise intestinal nutrient absorption, leading to such clinical manifestations (1, 45). The diverse internal and external parasites to which pigeons are vulnerable detrimentally affect their health and may culminate in death (47).

This investigation identified seven helminth species parasitising pigeons: three cestodes (*R. tetragona, R. echinobothrida* and *C. digonopora*), three nematodes (*A. galli, D. nasutus* and *Gongylonema* sp.) and one trematode (*Brachylaima* sp.). Cestodes dominated the parasitic load, with a collective prevalence of 67%. This last result parallels studies from Greece presenting 70.6% (13), India with 60% (26) and Pakistan indicating 60% (49), but contrasts with a lower rate in Egypt of 7.3% (14) and in Iran of 20% (41). The trematode *Brachylaima* sp. (5%) and nematodes *A. galli* (5%), *D. nasutus* (1%) and *Gongylonema* sp. (1%) exhibited the lowest prevalence. These findings align with trematode records from Egypt, where 0.14% trematode prevalence was reported (14), and Switzerland, where 2.9% was established (18), as well as with nematode prevalence in India of 5% (29) and in Pakistan of 6.7% (26).

The high prevalence of cestode infections in the examined pigeons likely stems from dietary exposure to intermediate invertebrate hosts. While pigeons predominantly consume grains and seeds, their opportunistic feeding behaviour may include taking invertebrates such as beetles, ants and termites, which serve as intermediate or reservoir hosts for cestodes (4, 7). In contrast, the sporadic occurrence of trematode infections raises questions about transmission pathways, as trematodes typically require freshwater snails as intermediate hosts (12), for which the habitats are notably scarce in the arid UAE environment. Some artificial freshwater lakes have been constructed in the country and have abundant populations of *Melanoides* spp. snails (15); these could provide ample opportunities for some trematode life cycles to persist. While *Brachylaima* species circumvent this by utilising terrestrial snails and slugs as intermediate hosts (48), the UAE’s environment similarly fails to sustain significant populations of these organisms, complicating explanations for infection pathways. Surprisingly, nematodes exhibited low prevalence despite their frequent dominance in other regions, where they are often transmitted *via* contaminated food or water (40, 43). This discrepancy may reflect limited environmental suitability for nematode transmission in the UAE. Collectively, the reduced prevalence of both nematodes and trematodes may be attributed to the region’s extreme climatic conditions, where elevated temperatures and aridity likely hinder the survival, development and dispersal of their infective stages.

Single-species helminth infections predominated over mixed infections, a pattern consistent with some prior findings (13, 31) but also one contrasting with earlier reports of higher mixed-infection rates (6). The dominance of single infections may reflect ecological competition among parasites: initial colonisers often monopolise microhabitats within the host, limiting opportunities for subsequent species to establish themselves (28). Additionally, host dietary habits may shape parasite community structure, as resource availability and foraging behaviour influence exposure to specific parasite taxa (24, 42). These factors suggest that infection patterns arise from a complex interplay of parasite interactions and host ecology.

This study revealed a higher prevalence of helminth infections in male pigeons (71.4%) than in females (65.9%), aligning with prior studies indicating greater male susceptibility to parasitic infections (31, 49). Such disparities may stem from intrinsic biological differences between sexes that influence host–parasite interactions (36). Behavioural patterns and physiological responses, such as males’ broader roaming ranges or females’ exposure to physiological stress during brooding periods, may further contribute to infection susceptibility. Elevated infection rates in females could also reflect stress-related impacts on immune function, as parasite development often correlates with host stress levels and immune resilience (23, 46). However, some studies suggest that sex may not be a decisive factor in infection risk (3, 26), highlighting the need for context-specific analyses.

Notably, feral pigeons exhibited substantially higher helminth infection rates (92%) than their domestic counterparts (46%). This divergence likely arises from ecological and management factors: feral populations occupy diverse environments and move around unrestrictedly (39), increasing their exposure to pathogens, and they also receive no veterinary care nor are managed for good health. In contrast, domestic pigeons benefit from human intervention, including preventive healthcare and regulated living conditions, which mitigate infection risks.

The study also observed elevated helminth infection rates in adult pigeons, which were likely to be the results of prolonged environmental exposure. Adult birds’ capacity for long-distance flight and access to diverse habitats may increase interaction with infected conspecifics or contaminated environments, thereby amplifying transmission opportunities. In contrast, earlier studies reported higher infection prevalence in juvenile birds, potentially a consequence of their underdeveloped immune defences (34, 38).

## Conclusion

While this study provides foundational data on helminth infections in pigeons in Al Ain, several considerations warrant attention to guide future research. The focus on adult pigeons offers insight into the longer-term accumulation of parasites, but inclusion of juvenile birds in future work could help clarify age-related susceptibility patterns. Although clinical signs such as diarrhoea and weight loss were noted in infected individuals, future studies could quantify these effects more precisely and explore correlations with parasite burden. The identified helminths include species with potential zoonotic significance, particularly in urban settings, and further investigation into public health implications is encouraged. While seasonal trends were not specifically analysed in this study, the extended sampling period provides a representative overview and lays the groundwork for more targeted temporal analyses. Lastly, the use of necropsy-based identification ensured accurate parasite detection, offering a robust baseline for comparison, even as methodological differences across studies remain an important consideration in interpreting prevalence variations.
